# Tumors of the reproductive tract of sheep and goats: A review of the current literature and a report of vaginal fibroma in an Awassi ewe

**DOI:** 10.14202/vetworld.2019.778-782

**Published:** 2019-06-11

**Authors:** Wael M. Hananeh, Zuhair B. Ismail, Mousa H. Daradka

**Affiliations:** 1 Department of Pathology and Public Health, Faculty of Veterinary Medicine, Jordan University of Science and Technology, Irbid 22110, Jordan; 2 Department of Clinical Veterinary Sciences, Faculty of Veterinary Medicine, Jordan University of Science and Technology, Irbid 22110, Jordan

**Keywords:** benign tumors, malignancy, reproductive tract, small ruminants

## Abstract

**Aims:**

This study aimed to provide a summary of all online available literature of published clinical and histopathological data regarding tumors affecting the reproductive tract of female sheep and goats. In addition, a detailed description of the clinical history, clinical findings, and gross and histopathological findings of one case of vaginal fibroma in an adult Awassi sheep is provided for the first time.

**Materials and Methods:**

Internet search engines such as PubMed, ResearchGate, Scopus, ScienceDirect, and Google Scholar were used to collect all published articles in refereed journals from 2000 to 2018 regarding tumors and tumor-like lesions involving the reproductive tract of ewes and does.

**Results:**

There are six published papers in sheep and nine in goats reporting various malignant and non-malignant tumors involving different parts of the reproductive tract. The most commonly diagnosed tumors of the reproductive tract were leiomyoma (six cases), adenocarcinoma (six cases), leiomyosarcoma (three cases), adenoma (one case), squamous cell carcinoma (one case), and metastatic signet ring cell carcinoma (one case). The most common sites of tumor formation were the uterus (10 cases), vulva/vagina (five cases), ovaries (four cases), cervix (four cases), and Bartholin’s gland (one case). All affected animals were aged females (older than 3 years of age). In both ewes and does, the most frequently reported clinical symptoms were chronic weight loss, pyometra, hydrometra, vaginal bleeding, abnormal vaginal discharges, straining, pollakisurie, ascites, and abdominal distension.

**Conclusions:**

Tumors of the reproductive organs in sheep and goats are not uncommon and should be considered in the differential diagnoses in cases with poor reproductive function.

## Introduction

Malignant and non-malignant tumors of the female reproductive tract in small ruminants are not uncommon and rank in frequency only second to skin tumors [1]. Approximately 10-50% of such tumors are of smooth muscle origin [1-3]. Tumors of the reproductive organs can be found affecting the vulva, vagina, cervix, uterus, and ovaries with varying degrees of malignancy [1-3].

These tumors could lead to a significant economic loss due to infertility, production losses, and the death of the affected animals in advanced cases [1-3]. Nevertheless, early diagnosis and surgical excision could be curative in some cases [1]. Data regarding the clinical, histopathological, and surgical excision of various types of tumors of the reproductive tract in sheep and goats are scattered and relatively scarce in recent literature.

This study aimed to summarize the clinical and histopathological data regarding tumors affecting the reproductive tract of female sheep and goats that have been published in scientific and refereed journals. In addition, a detailed description of the clinical history, clinical findings, and gross and histopathological findings of one case of vaginal fibroma in an adult Awassi sheep is provided for the 1^st^ time.

## Materials and Methods

### Ethical approval

Written owner consent was obtained before the use of the clinical case described here in this article. Institutional Animal Ethic Committee approval was not required as the study was based on clinical case.

### Database search

A review of recent literature regarding malignant and non-malignant tumors involving the reproductive organs in female sheep and goats was carried out using internet search engines such as PubMed, ResearchGate, Scopus, ScienceDirect, and Google Scholar. Only published papers in scientific and refereed journals after the year 2000 to 2018 were considered. Keywords that were used in the search included “tumors of the reproductive tract in sheep,” “tumors of reproductive tract in goats,” “tumors of the vulva and vagina in sheep and goats,” “tumors of the cervix and uterus in sheep and goats,” and “tumors of the ovaries in sheep and goats.” To avoid missing any papers, the references of all selected articles were also searched.

### Data extraction

Data extracted from selected articles included the species, breed, gender and age of affected animals, histopathological diagnosis, location of the lesion, and clinical history wherever specified.

## Results

**Figure-1:**
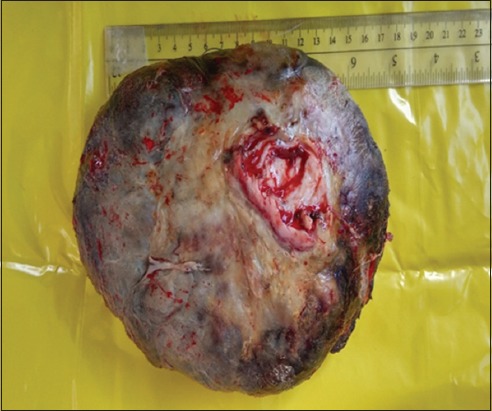
A discrete, large (19×14×10 cm), sessile, firm reddish fibroma surgically removed from the vagina of a 3-year-old female Awassi sheep.

**Figure-2:**
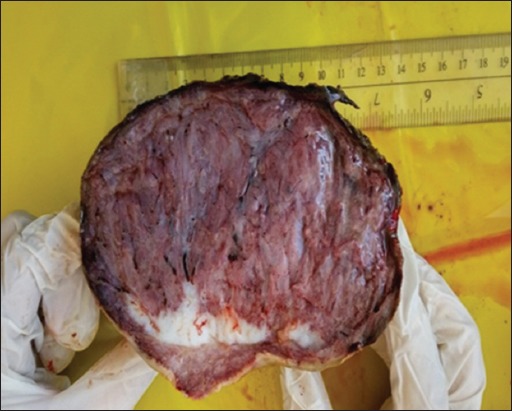
Cross-section of the vaginal fibroma showing fleshy and distinctive fascicula appearance.

**Figure-3:**
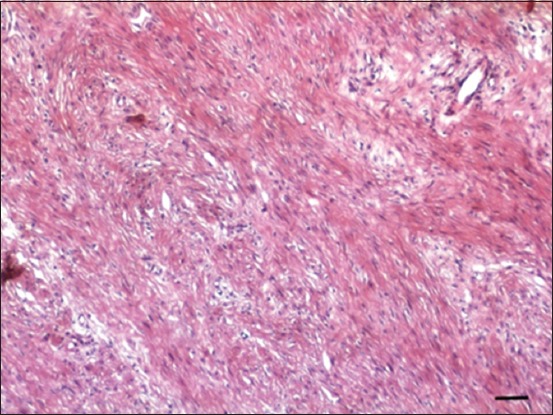
Hematoxylin and eosin stained section of the fibroma showing streams and bundles of well-differentiated spindle connective tissue cells that are haphazardly arranged and running in different directions with abundant collagen (Bar=50 µm).

**Figure-4:**
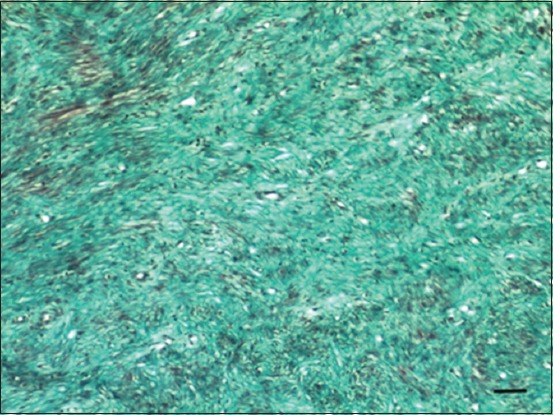
Masson’s trichrome stained sections using light green of the vaginal fibroma showing greenish-colored collagen bundles that are running in different directions (Bar=50 µm).

Review of recent literature revealed five published papers in sheep [4-9] (Table-1) and eight in goats [3,8,10-16] (Table-2) reporting various malignant and non-malignant tumors involving different parts of the reproductive tract [3-8,10-15,17-20]. Parts of the reproductive tract that was involved included the vulva and vagina (two in sheep, three in goats), Bartholin’s gland (one goat), cervix (one in sheep, three in goats), uterus (two in sheep, nine in goats), and ovaries (two in sheep, two in goats). In sheep, the most common tumors of the reproductive organs were granulosa cell tumors (GCTs) (two cases), leiomyoma (one case), leiomyosarcoma (one case), squamous cell carcinoma (SCC) (one case), and fibroma (one case). All sheep were aged ewes (older than 3 years of age). The most frequently reported clinical symptoms in sheep were infertility, weight loss, pyometra, and vaginal bleeding. In this article, description of the clinical history, gross and histopathological findings of an intravaginal fibroma was described for the first time in a 3-year-old Awassi ewe. The ewe was presented for evaluation of a large vaginal mass of unknown duration. The mass was surgically removed and submitted for histopathological evaluation. The mass was large (19×14×10 cm), ovoid in shape and sessile, firm, and reddish in color (Figure-1). On cross section, the mass was fleshy and exhibited fascicula appearance (Figure-2). Histologically, the mass was completely encapsulated and consisted of well-differentiated densely packed neoplastic cells supported by a fine vascular network. These neoplastic cells were spindle and forming streams and interwoven bundles. These streams and bundles were haphazardly arranged and running in different directions with abundant collagen (Figure-3). The neoplastic cells had abundant eosinophilic cytoplasm and a centrally located elongated basophilic nucleus. The mitotic figures were rare. To discriminate collagen fibers from muscular tissues on histological sections, Masson’s trichrome staining was used with light green. Throughout the section, the neoplastic cells exhibited a strong diffuse cytoplasmic greenish staining (Figure-4). Masson’s trichrome stain confirmed the diagnosis of fibroma.

**Table-1:** The signalment, tumor types, organs affected, and symptoms of tumors affecting the reproductive organs in sheep.

Reference	Tumor type	Organ(s)	Signalment	Symptoms
Corpa and Martinez [4]	Leiomyoma	Uterus	NS	NS
Vemireddi *et al*. [5]	Leiomyosarcoma	Uterus	3-year-old Suffolk ewe	Vaginal bleeding
Svara *et al*. [6]	Granulosa cell tumor	Ovary	5-year-old Pramanka cross ewe	Weight loss
Oultram and Morgan [7]	Granulosa cell tumor	Ovary	Aged pet ewe	Pyometra
Ferrer *et al*. [8]	Squamous cell carcinoma	Vagina	10-year-old Rasa Aragonesa ewe	Weight loss
				Infertility
		Cervix		
Verberckmoes *et al*. [9]	Fibroma	Vagina	12-year-old ewe	Straining

NS=Not specified

**Table-2:** The signalment, tumor types, organs affected, and symptoms of tumors affecting the reproductive organs in goats.

Reference	Tumor type	Organ(s)	Signalment	Symptoms
Whitney *et al*. [3]	Leiomyosarcoma	Uterus	12-year-old Saanen doe	Vaginal bleeding
		Uterus	13-year-old Saanen goat	pseudopregnancy
Kawashima *et al*. [10]	Adenocarcinoma	Uterus	11-year-old doe	Weight loss
Lohr [11]	Leiomyoma	Uterus	Diverse breeds with the majority dwarf, Nubian, and Saanen goats Median age 3.1 years	NS
		Uterus		
	Squamous cell carcinoma	Vagina		
		Vulva		
	Adenocarcinoma	Cervix		
		Uterus		
	Leiomyofibroma	Vagina		
		Cervix		
Pfisterl *et al*. [12]	Leiomyoma	Uterus	6-year-old dwarf doe	Pollakisurie Hydro pyometra
Dockweiler *et al*. [13]	Adenocarcinoma	Uterus	Mixed breed doe	Vaginal discharge
	Leiomyosarcoma	Uterus		
Uzal and Puschner [14]	Leiomyoma	Cervix	17-year-old Toggenburg doe	Severe hemorrhage and death
Ferrer *et al*. [8]	Adenoma	Bartholin’s gland	7-year-old Saanen doe	Enlarged vulva
Beena *et al*. [15]	Adenocarcinoma Thecoma	Ovary	NS	NS
Stern *et al*. [16]	Metastatic signet ring cell carcinoma	Ovary	3.5-year-old Boer doe	Abdominal distension

NS=Not specified

In goats, the most frequently diagnosed tumors of the reproductive organs were leiomyoma (five cases), adenocarcinoma (six cases), leiomyosarcoma (two cases), adenoma (one case), choriocarcinoma (one case), SCC (one case), thecoma (one case), leiomyofibroma (one case), and signet ring cell carcinoma (one case). All goats were females older than 3 years of age. The most frequent clinical symptoms were weight loss, vaginal bleeding, abnormal vaginal discharge, pollakisurie, hydro pyometra, and abdominal distension.

## Discussion

### Adenocarcinoma

Adenocarcinoma of the reproductive organs is rarely diagnosed in ruminants [2]. However, in this review, all adenocarcinomas were diagnosed in goats and none were found in sheep. Adenocarcinoma is considered highly metastatic and locally invasive [2]. In one goat, a combined pathology of uterine adenocarcinoma and choriocarcinoma was diagnosed. Metastatic tumorous cells were also found in the kidney, liver, and lung [10]. In a large study, involving 1146 necropsy or biopsy specimens from goats, two adenocarcinomas were diagnosed in the cervix or cervix and uterus, and one was in the vagina [11]. Metastatic lesions were also found in lungs and local lymph nodes [11]. In another study, a combined pathology consisted of uterine adenocarcinoma and leiomyosarcoma was diagnosed in an aged mixed-breed goat doe with a 9-month history of serosanguinous vaginal discharge [13]. Necropsy of this doe revealed multiple masses involving the uterus, cervix, and lung [13].

### Leiomyoma

Leiomyoma is a non-invasive benign tumor of the smooth muscles which has been rarely reported in ruminants, especially sheep and goats [2,4,17]. Leiomyomas are considered far more common than leiomyosarcoma [2]. Histologically, the tumor is consistent of neoplastic cells of smooth muscles with variable degrees of differentiation and variable quantities of connective tissue with no evidence of glandular tissues [2]. Grossly, the tumor may reach up to 12 cm in diameter. In the reproductive tract, the most common sites for leiomyoma formation are the uterus, vagina, and cervix [2]. Leiomyoma was diagnosed in the cervix in a 17-year-old Toggenburg doe with a history of acute vaginal bleeding and death [14]. The tumor was found to originate from the cervix and occupy most of the vaginal lumen [14]. Three cases of leiomyoma (two uterine, one vagina) were reported in a large study involving 1146 necropsy or biopsy specimens from goats [11]. Uterine leiomyoma was also diagnosed in a dwarf goat with a history of chronic pollakisurie, tenesmus, and hydrometra [12].

### Leiomyosarcoma

Leiomyosarcoma is locally invasive malignant tumor of smooth muscles that are capable of metastasis to distant locations in the body [2,17]. Most of the leiomyosarcomas are diagnosed incidentally in abattoirs [2]. The differentiation between leiomyoma and leiomyosarcoma is usually based on microscopic findings of increased mitotic figures, invasiveness, and presence or absence of necrosis [2]. In one case, uterine leiomyosarcoma was diagnosed and removed surgically from a 3-year-old Suffolk ewe [5]. The ewe suffered from vaginal bleeding, spontaneous lactation, and nursing behavior [5]. The tumor was multiple, well-circumscribed, intraluminal polypoid masses of variable sizes (0.5-4 cm) [5]. Histologically, neoplastic cells were characterized by the presence of basal lamina, scant microfilaments, contracted nuclei, and flat intercellular junctions [5]. In goats, leiomyosarcoma was diagnosed in two aged Saanen does (age 12 and 13 years) that suffered repeated episodes of pseudopregnancy and died due to severe hemorrhage from the vulva [3]. Histological evaluation of the vagina, cervix, and uterus revealed dense and pleomorphic spindle cells forming a dense collagenous stroma indicating a diagnosis of leiomyosarcoma [3]. In one goat, two types of tumor cells in the uterus consisting of adenocarcinoma and leiomyosarcoma were diagnosed [13]. The goat was an aged mixed breed doe with a 9-month history of serosanguineous vaginal discharge [13]. The case was characterized by the presence of multiple masses within the uterus, cervix, and lung [13]. Histological evaluation of the masses revealed mixed tumorous cells, indicating a collision tumor of primary adenocarcinoma and leiomyosarcoma [13].

Gastrointestinal stromal tumors (GISTs) that are mostly arise from the gastrointestinal tract can mimic leiomyomas and leiomyosarcomas [18]. GISTs have not been reported in reproductive tracts of small ruminants; however, a set of immunohistochemistry panel can be used to differentiate between these tumors. The neoplastic cells of leiomyomas and leiomyosarcomas are strongly and diffusely positive for smooth muscle actin and desmin but negative for CD34 and CD117 while GISTs are strongly and diffusely positive for CD117, with a cytoplasmic, membranous, or paranuclear “dot-like” pattern [19].

### GCT

GCT is one of the most common ovarian tumors in domestic animals [1-3]. The tumor is composed of neoplastic granulosa cells, theca cells, and f5 GCT is usually considered rarely metastatic and non-invasive [2]. There is no GCT reported in goats. In one sheep, malignant GCT was diagnosed in an adult ewe with severe and progressive weight loss [6]. In this case, multifocal metastatic lesions were found in the lung, spleen, mediastinal, and iliac lymph nodes [6]. In another case, GCT was diagnosed in a pet sheep with pyometra that was surgically removed with favorable outcome [7]. Adenocarcinoma and thecoma were diagnosed affecting the ovary in one 8-year-old goat [15]. Metastatic lesions were also found affecting the local lymph nodes, lungs, pleural cavity, and pericardium in this goat [15].

### SCC

SCC is a common cutaneous malignant tumor in all domestic animals that are characterized by tumorous epithelial cells with variable degrees of differentiation toward keratinocytes [2]. In one sheep, SCC was found affecting the vagina and cervix causing infertility [8]. The tumor was found invasive and histologically characterized by the presence of atypical squamous epithelial cells, round anaplastic cells, necrosis, keratinization, and infiltration by mononuclear cells [8]. In goats, one SCC was also diagnosed affecting the vulva [11].

### Adenoma

Tumors of the Bartholin’s gland are rare in animals [2]. Tumors of the Bartholin’s gland are usually classified as adenomas and carcinomas, with most of the tumors arising from either the mucin-secreting columnar epithelial cells or the squamous epithelium at the vestibular orifice of the gland [2]. In this review, only one case of adenoma of the Bartholin’s gland was diagnosed in a goat [12]. The doe was a 7-year-old pregnant Saanen goat with bilateral enlargement of the vulva [12]. Grossly, the tumor was involving the vulva with multilobulated cystic masses [12]. On histological evaluation, the tumor was characterized by proliferation of irregularly shaped, glandulotubular neoplastic epithelial cells [12].

### Fibroma

Fibroma and leiomyofibroma are benign tumors of the fibrous connective tissue and smooth muscles, respectively [2]. Fibroma of the reproductive tract is rarely reported in small ruminants. Leiomyofibroma was diagnosed in affecting the cervix in one goat [11]. In most recent literature, vaginal fibroma was reported only once in a 12-year-old pregnant crossbreed Texel ewe [9]; however, in this case, we report a vaginal fibroma in a 3-year-old non-pregnant Awassi sheep. Furthermore, the tumor size in Awassi sheep was much bigger than that found in crossbred Texel sheep. The mass size measured (19×14×10 cm) in Awassi sheep while it was the size of a fist in the Texel ewe. In Awassi sheep, the entire mass was surgically excised and sent for pathological examination while in Texel sheep pathological diagnosis did depend on a tumor biopsy by means of biopsy needle. Both animals showed similar histopathological features.

### Signet ring cell carcinoma

Metastatic signet ring cell carcinoma is extremely rare in animals. In humans, only few cases have been reported [16]. These tumors are usually characterized by its low invasiveness and non-aggressive nature. In one Boer goat, metastatic rather than primary ovarian signet ring cell carcinoma was considered due to the presence of many neoplastic cells spread throughout the abdominal cavity [16]. Extensive infiltration of the colon with signet ring cells indicated the gastrointestinal tract as the primary site of the tumor in this case [16]. Clinical symptoms in the goat with the metastatic ovarian signet ring cell carcinoma were acute recumbency and ascites [16].

## Conclusion

Although rarely diagnosed in small ruminants, tumors and tumor-like lesions still can be encountered in veterinary practices worldwide and should be considered in the differential diagnoses of infertility and abnormal reproductive function in sheep and goats. This review indicates that goats are more likely to suffer from tumors involving the reproductive tract than sheep. In addition, a case of vaginal fibroma in a non-pregnant Awassi sheep is described here for the 1^st^ time.

## Authors’ Contributions

WMH diagnosed the case and wrote the pathology part of the manuscript. ZBI completed the rest of the manuscript. MHD did the clinical and surgery part. All authors read, finalized, and approved the manuscript.
